# Evaluating the Number of Stages in Development of Squamous Cell and Adenocarcinomas across Cancer Sites Using Human Population-Based Cancer Modeling

**DOI:** 10.1371/journal.pone.0037430

**Published:** 2012-05-22

**Authors:** Julia Kravchenko, Igor Akushevich, Amy P. Abernethy, H. Kim Lyerly

**Affiliations:** 1 Duke Cancer Institute, Duke University Medical Center, Durham, North Carolina, United States of America; 2 Center for Population Health and Aging, Duke University Medical Center, Durham, North Carolina, United States of America; 3 Department of Medicine, Division of Medical Oncology, Duke University Medical Center, Durham, North Carolina, United States of America; 4 Department of Surgery, Division of General Surgery, Duke University Medical Center, Durham, North Carolina, United States of America; 5 Department of Pathology, Duke University Medical Center, Durham, North Carolina, United States of America; Vanderbilt University Medical Center, United States of America

## Abstract

**Background:**

Adenocarcinomas (ACs) and squamous cell carcinomas (SCCs) differ by clinical and molecular characteristics. We evaluated the characteristics of carcinogenesis by modeling the age patterns of incidence rates of ACs and SCCs of various organs to test whether these characteristics differed between cancer subtypes.

**Methodology/Principal Findings:**

Histotype-specific incidence rates of 14 ACs and 12 SCCs from the SEER Registry (1973–2003) were analyzed by fitting several biologically motivated models to observed age patterns. A frailty model with the Weibull baseline was applied to each age pattern to provide the best fit for the majority of cancers. For each cancer, model parameters describing the underlying mechanisms of carcinogenesis including the number of stages occurring during an individual’s life and leading to cancer (*m*-stages) were estimated. For sensitivity analysis, the age-period-cohort model was incorporated into the carcinogenesis model to test the stability of the estimates. For the majority of studied cancers, the numbers of *m*-stages were similar within each group (i.e., AC and SCC). When cancers of the same organs were compared (i.e., lung, esophagus, and cervix uteri), the number of *m*-stages were more strongly associated with the AC/SCC subtype than with the organ: 9.79±0.09, 9.93±0.19 and 8.80±0.10 for lung, esophagus, and cervical ACs, compared to 11.41±0.10, 12.86±0.34 and 12.01±0.51 for SCCs of the respective organs (p<0.05 between subtypes). Most SCCs had more than ten *m*-stages while ACs had fewer than ten *m*-stages. The sensitivity analyses of the model parameters demonstrated the stability of the obtained estimates.

**Conclusions/Significance:**

A model containing parameters capable of representing the number of stages of cancer development occurring during individual’s life was applied to the large population data on incidence of ACs and SCCs. The model revealed that the number of *m*-stages differed by cancer subtype being more strongly associated with ACs/SCCs histotype than with organ/site.

## Introduction

Multiple studies have demonstrated that adenocarcinomas (ACs) and squamous cell carcinomas (SCCs) of the same organs (such as lung, esophagus, and cervix uteri) differ by the role that various risk factors play (e.g., smoking, body mass index and body fat distribution, HPV subtypes, etc.) as well as by their clinical presentations (e.g., patients with cervical and lung AC have poorer prognoses, higher stromal invasion, metastasized more easily, and are more resistant to radiotherapy than patients with cervical and lung SCC) [1]–[12].On the molecular level, differences between ACs and SCCs have been also observed. For example, more genetic changes have been found to accumulate in SCCs, tumor suppressor genes for these two subtypes are located on different chromosomes, and ACs differed from SCCs by the levels of expression of apoptosis inhibiting factor (i.e., survivin) and tumor-invasion related factor (i.e., matrix metalloproteinase-2 and -7) [13]. Based on similarity of age-incidence patterns identified on logarithmically scaled plots, it has been suggested that tumors which had common embryonic cellular ancestry, differentiation pathways, and histologic characteristics may have similar characteristics related to carcinogenesis processes even when arising from different organs [14], [15].

If similarities exist between ACs and SCCs in clinical and molecular studies, then certain similarities within histotypes may also exist for underlying mechanisms of carcinogenesis. For example, when supposed that a population of cells must experience a number of stochastic events (*m*-stages) in the path toward a clinically diagnosed cancer, then the numbers of such events may differ for ACs and SCCs. We hypothesized that certain similarities may exist between characteristics related to carcinogenesis for ACs and for SCCs that could be even stronger than organ-specific similarities. To test this hypothesis, we evaluated tumor characteristics by applying a model to describe the age patterns of incidence rates of ACs and SCCs across cancer sites using the data from the large cancer registry. Our approach was based on the idea that patients have to pass a certain number of stages on their way to clinically diagnosed cancer. The current understanding of these stages is more general than in the majority of existing models of carcinogenesis, which assume sequential mutations are the main driving forces of carcinogenesis. In our model, the person (not the cell) has to pass from stage to stage; at certain stages individual states can be associated with mutations in susceptible cells. Rates of individual transitions between states are not the same for all individuals. Instead, we assumed that these rates were distributed in the population and parameters of this distribution were the subjects for estimation. Variance in these rates reflects variations in predisposition to certain cancers in population. In this framework, the number of unobserved stages is a model parameter (*m*-stages) that can be estimated by applying the model to human population data on cancer incidence. Our primary research task was to compare the estimates of *m*-stages for ACs and SCCs across cancer sites and find some regularity in the spectrum of found estimates. This modeling framework captures the base features of carcinogenesis that correspond to the chosen level of carcinogenesis simplification and allows for investigating the research questions of interest. However, that was not the only motivation of why this type of model was applied for analysis. Another reason was that our preliminary analyses [16] showed that this type of model provided a much better description of the age patterns of the incidence rate for majority of cancers in the US population up to the age of 85 years. In this paper, we demonstrate the ability of the model to describe age-patterns of incidence across a broad range of cancer sites. In spite of a good description of data on cancer incidence by the model, the risk of model misspecifications needs to be controlled further by detailed sensitivity studies that allow for testing for the stability of the results. The effects of trends in the stage at diagnoses, gender and racial differences, and age-period-cohort (APC) effects are incorporated into our base model and are in focus of our sensitivity studies.

## Materials and Methods

The age-adjusted incidence rates of fourteen ACs (lung, esophagus, stomach, colon, rectum, pancreas, liver, breast ductal, breast lobular, corpus uteri, cervix uteri, prostate, kidney, and ovary) and twelve SCCs (lung, esophagus, cervix uteri, larynx, anal, vulvar, lip, tongue, floor of mouth, gum and other mouth, tonsil, and hypopharynx) were analyzed over a 31-year period (1973–2003). The ACs and SCCs, which had more than 5,000 cases, were obtained from the list at the SEER Site Recode ICD-O-2 (at http://seer.cancer.gov/siterecode/icdo2_d01272003) for our defined time period (Table 1). For lung cancer, ACs (code 814) and SCCs (code 807) were selected as the most prevalent among those affecting the lung and having distinct clinical, pathological, and molecular characteristics. Thyroid AC was excluded from this analysis because of its numerous subtypes with unusual age distributions. The frequencies of the specific stage at cancer diagnosis (such as *in situ*, localized, regional, distant, and unstaged) were analyzed to determine the possible contribution of these changes to the characteristics of carcinogenesis for each studied cancer. Comparisons of age patterns of incidence for studied cancers diagnosed at all stages jointly and for invasive cancers alone were also performed.

**Table 1:** The frequencies of stages at cancer diagnosis in the SEER Registry, 1973–2003, in percent. (Initial, final, and years of significant changes – if needed - in stages distribution are presented).

Cancer Site (code)	Year	In situ	Localized	Regional	Distant	Unstaged
**Lung SCC (807)**	1983^1^	0.4	20.9	40.7	24.1	14.0
	2003	0.3	24.6	46.5	24.1	4.5
**Lung AC (814)**	1983^1^	–	20.4	34.4	37.2	8
	2003	–	19.5	38.0	38.8	3.7
**Stomach AC (814, 849)**	1973	0.3	15.9	33.1	32.6	18.2
	2003	0.9	22.0	33.0	34.5	9.6
**Esophagus SCC (807)**	1973	0.2	27.6	20.9	22.6	28.6
	2003	0.8	23.5	33.0	26.2	16.5
**Esophagus AC (814)**	1973	–	12.5	22.5	47.5	17.5
	2003	2	23.7	30.5	31.6	12.1
**Colon AC (814, 826, 848, 801, 821)**	1973	2.5	27.5	31.8	20.8	17.4
	2003	6.2	38.0	34.8	17.6	3.4
**Rectum AC (814, 826, 848, 821)**	1973	4	37.6	26.1	15.7	16.6
	2003	6.9	43.3	30.3	14.0	5.5
**Pancreas AC (814, 801, 848)**	1973	–	11.3	17.2	44.6	26.9
	2003	0.1	7.1	26.6	55.0	11.3
**Liver AC (817, 801, 814)**	1973	–	13.9	15.7	33.4	36.9
	2003	–	40.7	25.4	18.0	15.9
**Kidney AC (814, 831)**	1973	–	40.4	17.9	27.8	13.8
	2003	0.1	62.6	15.3	18.4	3.7
**Breast AC (850)**	1973	4.5	39.1	40.3	4.0	12.1
	1996	16.3	54.9	24.1	3.2	1.5
	2003	19	50.6	26.2	3.3	1.0
**Breast AC (852)**	1973	26.4	32.8	25.6	4.2	10.9
	2003	18	47.6	29.5	4.2	0.8
**Prostate AC (814)**	1983^1^	–	75.0		17.9	7.0
	2003	–	94.0		3.4	2.6
**Ovarian AC (814, 826, 838, 844, 846, 847)**	1973	–	24.6	7.7	54.5	13.2
	2003	0.4	14.5	6.6	76.6	2.0
**Corpus uteri AC (814, 838)**	1973	7.3	69.7	3.9	3.7	15.4
	2003	1.5	74.3	15.8	4.8	3.7
**Cervix uteri AC (814)**	1973	2.4	44.7	24.7	8.2	20.0
	1995	55.2	31.4	7.6	1.5	4.1
	1996	–	62.7	22.3	7.7	7.3
	2003	–	62.4	22.0	8.7	6.9
**Cervix uteri SCC (807)**	1973	63.2	19.6	9.5	2.5	5.2
	1995	88.2	6.2	4.1	0.7	0.8
	1996	–	52.1	36.3	6.6	5.0
	2003	–	47.3	40.1	9.3	3.3
**Larynx SCC (807)**	1973	3	52.9	25.3	3.0	12.5
	2003	5.9	41.5	46.5	3.5	2.6
**Anal SCC (807)**	1973	6.4	46.8	31.9	4.3	10.6
	2003	29.2	36.3	22.4	6.9	5.2
**Vulvar SCC (807)**	1973	20.5	43.9	19.7	1.5	14.4
	2003	63.5	20.8	10.8	1.3	1.8
**Lip SCC (807)**	1973	1.4	65.5	7.4	0.8	24.9
	2003	6.6	75.3	12.6	1.1	4.4
**Tongue SCC (807)**	1973	1.0	32.8	36.1	11.7	18.4
	2003	2.8	34.4	49.1	10.7	2.9
**Floor of mouth SCC (807)**	1973	1.6	29.5	41.0	8.2	19.7
	2003	6.2	38.1	46.0	5.2	4.5
**Gum and other mouth SCC (807)**	1973	1.2	35.6	36.4	10.7	16.2
	2003	3.8	34.3	49.7	6.2	6.0
**Tonsil SCC (807)**	1973	2.3	15.4	46.3	21.7	14.3
	2003	1.0	12.8	73.1	10.3	2.8
**Hypopharynx SCC (807)**	1973	–	17.1	40.3	27.1	15.5
	2003	0.8	11.6	66.9	17.2	3.4

Notes: * Only for prostate cancer (1983–2003 all localized and regional cases coded as “Localized/regional Prostate cases”.^1^– Data on stages prevalence are available since 1983.

The age patterns of incidence rates for the fourteen ACs and twelve SCCs were studied for quality of fit for various models. We considered the one-year age interval for age-specific incidence rates. Two-stage modeling approach was applied for the spectrum of these age patterns. Analysis at the first stage was designed to select the best model by applying the known carcinogenesis models to sex-, race-, and year-specific age patterns. At the second stage, the best model for ACs and SCCs was generalized to analyze the data independent of sex-, race- and year-specificity.

To diminish the effects of advances in screening and diagnostics, we analyzed cancer incidence rates for three periods (1973–1983, 1984–1993, and 1994–2003) (see Table S1). The classic Armitage-Doll model [17], the two-stage clonal expansion (TSCE) model, and several types of the models with hidden frailty were tested. Our analysis confirmed the conclusions made by evaluating the quality of fit of each model to all sex-, race-, and time period specific age-patterns of ACs and SCCs using χ^2^ and Fisher’s criteria, that the frailty model with the Weibull baseline with the frailty described by a family of distributions (gamma or inverse Gaussian) provided the best fit for majority of cancers [16]. First, we applied this approach to an extended set of race-sex-time period-specific analyses of 264 age patterns of cancer incidence (Table S1). The analytic expression of the model for incidence rate is:



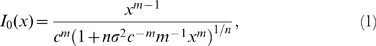
where: *x* is the age at cancer diagnosis, *m* (*m*-stages) is the number of stages occurring during the person’s life and leading to cancer development, *c* (in years) is the parameter related to the maximum age in the cancer incidence age pattern, 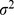 is the variance of the frailty distribution that reflects an individual susceptibility to cancer risk, and n describes the shape of the frailty distribution (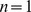, 2, and 0 corresponds to gamma-distribution, inverse Gaussian distribution, and the distribution suggested in Manton et al. [18] respectively). For 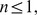 the shape of the age-pattern represented by the model has a maximum with age equal to 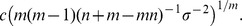 In our model, the term “*m*-stages” describes the number of “***m***alignant” rate-limiting events that a person had on the way of to the occurrence of malignant tumor (the “*m*” was added before “stages” to distinguish from the “stages at diagnosis”); thus, the meaning of *m*-stages here does not correspond exactly to one from a classic work of Armitage-Doll [17] or from other models of carcinogenesis such as MVK [19], [20] and TSCE [19], [20]. Our goal was to compare all cancers simultaneously to test the general hypothesis about the differences between ACs and SCCs. To do so, we adopted a parsimonious style of modeling that resulted in minimal number of weakly correlated parameters.

For each cancer, the minimum age of cancer incidence patterns to be analyzed was selected based on the results of empirical analysis of the maximum age at which there were no cancer cases recorded in the SEER registry. The minimum age estimate was 30 years for cancers of lung, stomach, esophagus, colon, rectum, pancreas, liver, kidney, breast lobular, corpus uteri, and cervical AC, and 15 years for SCCs of cervix, anus, vulva, and head and neck. The highest minimal age was for patients diagnosed with prostate cancer (40 years old).

At the first stage of analysis, age-specific cancer rates were evaluated with the standard errors and age patterns were fitted by the model (1). For example, for patients with lung ACs, 55 age-specific rates (from ages 30 to 84 years old) were used. At the second stage, the model was generalized to analyze age-, year-, sex-, and race-specific patterns for each cancer site (for ACs and SCCs). For example, for lung AC, a total of 6820 patterns were fitted: i.e., 55 patterns [for 55 age-specific groups] x 31 patterns [for 31 years] x 2 patterns [for males and females] x 2 patterns [for Caucasians and African-Americans]. The generalized model is:



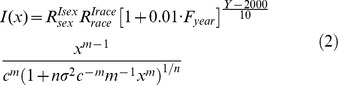
where 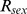 and 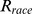
are relative risks of increased cancer incidence for females and for African-Americans, respectively (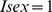 for female and 0 for male, and 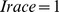 for African-Americans and 0 for Caucasians); 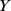 is a calendar year, and 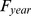 is related to the percent change in incidence rates for a 10-year period. Parameters were estimated using nonlinear regression, with weights reciprocal to the variance estimated using the generalized Wilson’s approach [21]. The accuracy of the description of AC and SCC-specific incidence age patterns was evaluated by the value of *χ2/d.o.f* and by analysis of residuals for each fit for normality, heteroscedasticity, and autocorrelation (using SAS, SAS Institute; Cary, NC, Proc Model).

This analytic approach permitted the use of all ages within the SEER registry in the analysis, including ages above 80 years old, where decrease of cancer incidence rates is observed for majority of cancers. Decreasing cancer incidence rates at advanced ages must be appropriately reflected in the successful carcinogenesis model; this phenomenon often cannot be handles by the models of this class (e.g., TSCE) or remains ignored by researchers [22]. The most popular explanation of the decline in incident rates at advanced ages is that it is caused by the hidden heterogeneity in individual predisposition to cancer. The potential sources of such heterogeneity include the different stages of diagnosed cancer with likely different shapes of incidence rates, different sub-histological forms of cancer, different race effects and effects of genetic predisposition, different contributions of environmental exposure, and different effects of cohort, period, or both due to time trends coming from the progress in medical technologies, screening, and variety of clinical interventions (see also discussion by Yashin et al. [23]). While these sources of heterogeneity in an individual predisposition to cancer can be taken into account using available data (i.e., racial, gender, and cohort/period effects), the majority (e.g., genetic effects or environmental exposure) have to be modeled stochastically. Our modeling strategy involved explicit modeling of the effects of the first type using available data and stochastic modeling of the second type effects. In particular, the stochastic model involves two parameters to represent a distribution of the individual predisposition remaining after explicit inclusion of the effects of first type. These parameters are 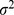 and 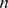. In model (2), racial, gender, and period effects were explicitly modeled. Because of parsimonious style of modeling, only one parameter is responsible for reflecting a period effect. Since it can be not sufficient to represent the variety of period/cohort effects, in sensitivity studies we applied age-period-cohort (APC) modeling as incorporated into carcinogenesis model according Moolgavkar et al. [24]. In this approach, period and cohort effects are represented non-parametrically. Specifically, the APC model linked to carcinogenesis model (2) is obtained by a substitution



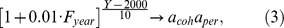
where cohort- and period-specific parameters 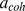 and 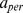 are subject for estimation.

## Results

**Figure 1:**
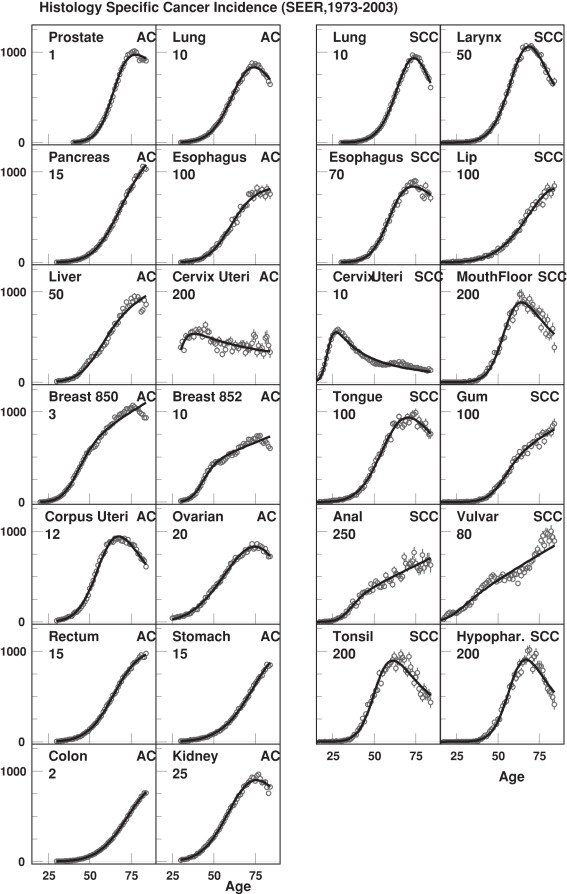
The results of model fitting for ACs/SCCs for each cancer site. (Rates for different cancers are rescaled to use the same scale on all plots for comparison. The original rate can be calculated by dividing the values obtained from the plot to the rescaled factor).

We applied mathematical models (1) and (2) to the SEER dataset. Model (1) was applied for sex-, race-, and decade-specific data (see Table S1). The main parameters characterizing carcinogenesis, including the number of *m-*stages (*m*), the age of maximal risk of cancer incidence (*c*), standard deviation of frailty distribution (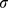), and the shape of the frailty distribution (*n*), did not vary substantially for most of the cancer sites by time period, sex, or race. There were, however, some visible trends for certain cancers. For example, there was a tendency for *m-*stages to decrease with time for cancers of head/neck, esophagus SCC, stomach, rectum, breast lobular, and prostate, and increase for lung SCCs. Males had slightly more m-stages than females for cancers of the stomach, colon, kidney, and tongue. Caucasians males with pancreatic cancer had slightly more *m*-stages than African-American males and slightly fewer *m*-stages in patients with laryngeal and tongue cancers. Caucasian females with rectal cancer had slightly fewer *m*-stages than African-American females. The majority of cancers had good model fit based on the *χ2/d.o.f* value, however, for prostate AC and cervical SCC *χ2/d.o.f* values where higher than required for good model fit (see Table 2). Among all the studied cancers, model (1) had a good fit with incidence patterns for a majority of AC and SCC; the fit was less precise for breast, cervical, and vulvar cancers (Figure 1). This discrepancy can be attributed to latent heterogeneity in these cancers that was not captured by the simple approach based on distributed frailty. For example, tumor grades and estrogen/progesterone receptor status can provide additional and significant contributions to such heterogeneity [25].

**Table 2:** The results of model fitting (presented as fitted parameters ±SE) for ACs and SCCs, SEER registry data, 1973–2003. (Parameters are summarized for both sexes and both races–see description of symbols used in headline in text).

Cancer	Age min, years	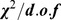	*c, years*	*m, number of m-stages*	*n*	*σ*	*R_sex_,*	*R_race,_*	*F_year_, %*
**Lung SCC**	30	2.71	79.1±0.3	11.41±0.10	0.57±0.03	7.3±0.1	0.25±0.00	1.33±0.02	−6.86±0.55
**Lung AC**	30	2.63	83.8±0.4	9.79±0.09	0.65±0.03	9.4±0.2	0.64±0.01	0.89±0.02	33.20±0.74
**Stomach AC**	30	1.09	100.4±1.3	8.28±0.14	1.79±0.16	11.6±1.1	0.37±0.00	1.03±0.02	−9.48±0.49
**Esophagus SCC**	30	1.07	89.6±1.2	12.86±0.34	0.83±0.04	30.1±1.3	0.30±0.01	2.75±0.08	−15.98±0.87
**Esophagus AC**	30	0.45	106.2±1.6	9.93±0.19	1.05±0.04	40.2±1.8	0.10±0.00	0.10±0.00	151.85±2.70
**Colon AC**	30	1.65	83.4±0.3	7.99±0.05	1.23±0.10	3.8±0.1	0.74±0.00	1.07±0.01	−6.17±0.22
**Rectum AC**	30	1.14	85.2±0.7	9.67±0.13	1.36±0.03	14.2±0.5	0.55±0.00	0.42±0.01	−7.28±0.42
**Pancreas AC**	30	1.07	89.2±0.8	10.02±0.14	1.42±0.04	14.9±0.7	0.73±0.01	0.94±0.02	−3.41±0.45
**Liver AC**	30	1.01	99.6±2.0	9.76±0.25	1.09±0.07	29.9±2.2	0.23±0.00	0.92±0.03	85.55±2.34
**Kidney AC**	30	1.14	97.3±0.8	7.57±0.08	0.75±0.04	12.1±0.3	0.46±0.00	0.62±0.01	36.30±0.73
**Breast AC 850**	20	3.28	58.3±0.3	8.64±0.08	1.25±0.01	8.7±0.1	1.00±0.00	0.82±0.01	22.36±0.36
**Breast AC 852**	30	1.82	64.1±0.7	11.63±0.24	1.16±0.00	24.9±0.6	1.00±0.00	0.41±0.01	71.22±1.01
**Prostate AC**	40	15.23	67.5±0.2	14.08±0.16	0.87±0.02	4.5±0.1	1.00±0.00	1.55±0.03	43.75±1.05
**Ovarian AC**	25	1.39	106.0±1.6	5.95±0.09	0.73±0.07	10.8±0.4	1.00±0.00	0.30±0.01	−1.39±0.56
**Corpus uteri AC**	30	1.79	78.7±0.5	8.80±0.10	0.73±0.02	9.6±0.2	1.00±0.00	0.24±0.01	−7.13±0.49
**Cervix uteri SCC**	15	9.48	37.9±0.8	12.01±0.51	0.96±0.01	30.4±1.0	1.00±0.00	0.97±0.04	−49.99±0.64
**Cervix uteri AC**	30	0.77	74.2±10.6	8.42±1.38	0.90±0.03	100.5±10.9	1.00±0.00	0.15±0.03	36.99±2.57
**Larynx SCC**	15	0.87	81.7±0.5	11.09±0.13	0.83±0.02	17.2±0.3	0.12±0.00	1.04±0.02	−6.71±0.54
**Anal SCC**	15	0.44	91.8±5.5	11.96±0.85	1.12±0.01	272.5±21.9	1.36±0.04	0.35±0.03	279.69±15.02
**Vulvar SCC**	15	0.94	106.4±7.0	6.02±0.28	1.47±0.03	73.8±5.2	1.00±0.00	0.37±0.02	96.00±2.43
**Lip SCC**	15	0.42	132.3±1.2	7.15±0.07	0.00±0.00	14.2±0.2	0.10±0.00	0.10±0.00	−30.12±0.57
**Tongue SCC**	15	0.73	99.4±1.3	8.89±0.16	0.72±0.03	26.7±0.8	0.31±0.01	0.43±0.02	29.52±1.22
**Floor of mouth SCC**	15	0.49	89.8±1.3	11.97±0.29	0.71±0.03	44.4±1.3	0.24±0.01	0.53±0.04	−19.79±0.87
**Gum/other mouth SCC**	15	0.58	96.0±1.8	11.34±0.32	1.00±0.04	43.6±2.2	0.44±0.01	0.47±0.03	−5.05±1.02
**Tonsil SCC**	15	0.58	96.2±1.5	10.09±0.21	0.65±0.03	45.3±1.4	0.18±0.01	0.77±0.04	47.37±2.55
**Hypopharynx SCC**	15	0.44	98.9±1.3	11.21±0.22	0.47±0.04	35.2±1.0	0.12±0.00	0.74±0.04	−11.06±1.01

Notes: *c* - the generalized scale parameter of age dimension which characterizes the age of maximal incidence; *m* (*m*-stages) - the number of stages occurring during individual’s life and leading to the cancer diagnosis; *n* - the parameter running over different types of frailty distributions (e.g., *n* = 1 and *n* = 2 correspond to gamma-distribution and inverse Gaussian distribution); *σ* - characterizes the standard deviation of the frailty distribution (the distribution of cancer predisposition in population); *R_sex_* and *R_race_* describe the relative risks of cancer incidence in females and in African-American population, respectively; and 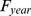 characterizes the percent change in cancer incidence rates for a 10-year period.

**Figure 2:**
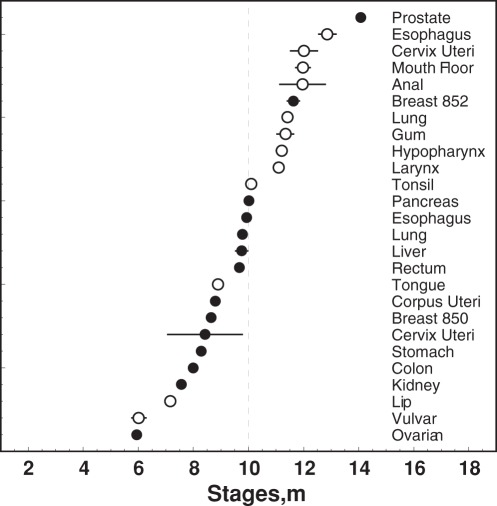
The estimated numbers of *m-*stages with respective standard errors (SE) for ACs (black dots) and SCCs (circles). Note: breast 850 – breast duct carcinoma, breast 852 – breast lobular carcinoma.

The number of *m*-stages determined using model (1) was the parameter of principal interest in this study (Table 2). There were no significant differences in the number of *m*-stages within either the ACs or within the SCCs groups. When cancers of the same organs were compared (i.e., ACs and SCCs of the lung, esophagus, and cervix uteri), the number of *m*-stages was similar within ACs (lung 9.79±0.09, esophagus 9.93±0.19, and cervical 8.80±0.10) and within SCCs (11.41±0.10, 12.86±0.34 and 12.01±0.51, respectively); the number of *m*-stages was greater for SCCs than for ACCs (p<0.05). This suggests that ACs and SCCs may require different numbers of events for cancer development. In general, SCCs appeared to require more *m-*stages for their development than ACs (Figure 2). Most SCCs had more than ten *m*-stages and ACs had fewer than ten *m*-stages, except for prostate and breast lobular cancers. The latter, probably, have two “forms”–younger” and “older”–that differ by patient’s age at manifestation, aggressiveness, response to treatment, and relation to sex hormone exposure. Recently, some of the contributing to such forms factors were studied for mechanisms of breast carcinogenesis [25].

To take into account the possible effects of sex, race, and time period, we included *R_sex_*, *R_race_* and *F_year_* parameters in a generalized model (2) (Table 2). The differences in parameters 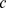 and 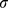 between cancers reflect the diversity of the respective incidence rates. Parameter 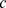
is the age dimension that characterizes the age at the cancer’s maximal incidence rate (existing for 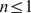), and 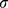 characterizes the shape of the distribution of predisposition to cancer in population (for distributions with large estimated values of 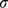 ^,^ the shape is largely concave, i.e., most individuals have a low predisposition, and the rest of the population is widely distributed). Parameters *R_sex_* and *R_race_* describe the relative risks of cancer incidence in females and in the African-American population, respectively. The strongest effect of sex (*R_sex_*≤0.30 in Table 2) was for cancers of the lung (SCC), esophagus (SCC and AC), liver, larynx, lip, floor of mouth, tonsil, and hypopharynx, while the strongest effect of race (*R_race_*≤0.30 in Table 2) was for esophageal AC, cervical AC, and cancers of ovary, corpus uteri, and lip, and for esophageal SCC (*R_race_* = 2.75). Parameter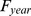 characterizes the percent change in incidence rate for a 10-year period in accordance with the results of empirical analysis of incidence trends: cancers of esophagus (AC), liver, breast lobular, cervix uteri (SCC), anal, and vulvar had the strongest effects of this parameter (absolute value of 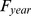
≥50%). The estimated values of *χ2/d.o.f* showed that the fit was improved when compared to the model (1) (see Table 2 and Supplemental Table S1).

Sensitivity analyses of the model parameters demonstrated the stability of the obtained estimates. Model parameters were not sensitive to a) the choice of the initial/minimal age at cancer diagnosis and inclusion/exclusion of the age group 85+ years old; b) the addition of a quadratic term describing time trends; c) the specific stratification of population groups (e.g., 5-year age interval); d) an estimation using the maximal likelihood approach rather the non-linear least squares; and e) considering specific time periods or stratifying population according to sex and race (Table S1). Also, the results did not significantly change when applying the APC to our model. For example, significant differences remained between numbers of *m*-stages for ACs and SCCs: for ACs and SCCs of the lung (8.90±0.13 and 9.84±0.13, p<0.05), esophagus (11.43±0.31 and 13.98±0.57, p<0.05), and cervical uteri (8.42±1.38 and 12.12±0.28, p<0.05), respectively. For the majority of cancers, parameters of the model did not change after incorporating the APC into the model; and the *m*-stage parameter was stable for all cancer sites. For some cancers (lung SCC, esophageal AC, breast lobular, and prostate) the estimated averaged numbers of *m*-stages shifted for about 1.5 stages, and for anal cancer it shifted even more. However, the direction of these shifts did not correlate with histotype (i.e., with ACs/SCCs tumor type).

Several interesting effects were observed during analysis of cohort- and period-specific parameters (i.e., 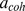 and 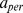 in Eq. (3)). For several cancers where birth cohort effects were observed, four different shapes of 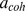 were evident: 1) increasing effect in older cohorts–for lung SCC and breast ductal carcinomas; 2) increasing effect in younger cohorts–for liver, breast lobular, and cervical SCC; 3) increasing effects till 1930–1939 birth cohort with subsequent decrease–for ACs of lung and corpus uteri; and 4) slightly decreasing effects for older cohorts with subsequent increase beginning from 1940–1949 birth cohort–for prostate cancer. The following calendar period effects represented by 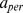 were also observed: 1) increasing with time effects–for ACs of lung, esophagus, liver, breast (both types), and kidney, and for anal, vulvar, tongue, and tonsil SCCs; 2) decreasing with time effects–for colon AC, and SCCs of lung and lip; 3) effects peaked around 1991–1995 years–for prostate AC and cervical SCC (likely, due to the introduction of active screening strategies at this time). In total, the results obtained from the main model demonstrated good stability after implementing the APC into the model.

## Discussion

In this study, the characteristics of carcinogenesis were analyzed across cancer sites and certain similarities were found inside cancer subtypes: adenocarcinomas (ACs) and squamous cell carcinomas (SCCs) likely require different numbers of stages for cancer development, with more *m-*stages required for SCCs than for ACs. In general, the obtained results confirmed out the hypothesis that characteristics of carcinogenesis may be more specific to cancer subtype (ACs or SCCs) than the organ/site. These results are consistent with other studies suggesting that oncogenesis could potentially be more informative when applied to distinct cancer subtypes rather than organs because their progression pathways may differ [15], [26], [27].

The results obtained in our study are also in agreement with multiple clinical observations on ACs and SCCs. For example, when ACs and SCCs of the same organs were compared (such as of lung, cervix uteri, esophagus, and gallbladder), patients with ACs had poorer prognosis and higher metastatic rates, and were more resistant to radiotherapy than patients with SCCs [4]–[6], [28], [29]. Effectiveness of chemotherapy were shown to differ for ACs and SCCs: e.g., a docetaxel (an anti-mitotic chemotherapy medication) was more effective in treatment of lung SCCs, while a pemetrexed (an antifolate antineoplastic agent) was more effective for lung ACs [30]. Different sets of immunohistochemical markers and their prognostics values have been identified for lung and cervical SCCs (such as higher expression of epidermal growth factor receptor, cyclin B1, p53, and COX-2) when compared with ACs (that had a higher expression of c-myc) [31]–[33]. Also, an increased expression of the embryonic stem cell gene set that is associated with poor survival has been observed for lung ACs but not for SCCs [34]. Altogether, these results affirm the differences in certain clinical characteristics and diagnostic markers between SCCs and ACs and agree with our findings that such differences could be more pronounced between histotypes than between tumors of different organs. Differences in characteristics of ACs and SCCs could be also illustrated (indirectly) by the studies on multiple primary cancers. Such studies demonstrate the frequent co-existence (at the same time or at separate times) of cancers of the same type (ACs or SCCs) at different locations in the same individual: e.g., for SCCs of the oral cavity and pharynx and esophagus, or of lip and skin; for ACs of the breast and ovary and corpus uteri, or of the prostate and urinary bladder, or of colon and rectum [35]–[37].

While our model was able to reveal the differences between ACs and SCCs, it could be capable to describe the differences between solid cancers (such as ACs and SCCs) and non-solid malignancies. We applied our model to the age patterns of incidence of leukemia and non-Hodgkin’s lymphoma from the SEER Registry data. It showed that leukemia (5.24±0.07) and non-Hodgkin’s lymphoma (4.29±0.04) had fewer *m*-stages than solid cancers such as ACs (7.8–9.8) and SCCs (10.2–13.8). The obtained results were in agreement with studies of other researchers that demonstrated that non-solid malignancies likely required fewer stochastic events/stages for their development than solid cancers [38], [39]. The latter often have pre-malignant lesions long before cancer is clinically diagnosed thus allowing us to hypothesize that some occult stages of solid cancer development result in larger number of stages occurring in individual.

The results obtained from our model showed that certain ACs such as lobular carcinoma of the breast or prostate cancer had more stages on average than the rest of ACs. When compared with other studies, our results were in agreement with their results demonstrating the differences, for example, between breast cancer and several other ACs: i.e., breast cancer differed in its somatic mutation spectrum from ACs of colon, rectum and pancreas leading to the conclusion that breast epithelial cells might be exposed to different levels or types of carcinogens or use distinctive repair systems [40], [41].

From the methodological point of view, our approach can be viewed in a historical perspective of developing carcinogenesis models that were applied to the age patterns of incidences of various cancers. It is still the subject for scientific debate on how to make a precise model and what kind of information could be obtained from them [42]. Armitage and Doll first demonstrated that age-mortality [17] and, later, age-incidence [43] patterns of certain epithelial cancers could be related to the number of cellular events (such as mutations) involved in the formation of a malignant tumor. Developed later by Moolgavkar and Knudson [44], and Tan [45] two-stage clonal expansion (TSCE) model and other multistage clonal expansion (MSCE) models, have different biological interpretations of the equivalent of the stages: e.g., in the TSCE model this parameter is closely related to promotion of pre-malignant cells. The understanding of *m*-stages in our model are not completely relevant to those above, as well as to those from the later generalizations of TSCE model capable for accounting for many sequential rounds of clonal expansion at different growth rates [46], [47]. Being a population-based, our model considers a person at a certain *m*-stage progressing to cancer onset. Transition of an individual from one *m*-stage to the next could be interpreted as a generalized “carcinogenic event” that occurred at a certain rate mathematically related to model parameters: i.e., parameter 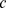 is related to averaged transition rate between *m*-stages, and parameters of frailty distribution (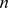 and 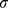) describe the distribution of this rate in population. The cancer-specific number of *m*-stages can be estimated as one of the model parameters allowing for comparison of *m*-stages among cancer site and their ACs/SCCs type. The transitions between *m*-stages in our model can be associated with mutations, adverse epigenetic or stromal events. However, the number of *m*-stages in our analysis is not fully corresponding to the number of oncogenic/molecular changes because several carcinogenic molecular changes could occur within the same *m*-stage [48]. Different molecular analyses suggested different numbers–from four to seven–of oncogenic molecular changes that may feature on colon cancer and at least ten–for prostate cancer [20], [46], [49]–[51]. Recent studies demonstrated that ACs and SCCs could differ by the involvement of different anti-cancer barriers. For example, the inhibition of apoptosis plays more important role in cervical ACs, while tumor-invasion related factors are more important for cervical SCCs [52]. To include molecular mechanisms in our carcinogenesis model, the concept of barrier mechanisms that was recently developed for non-solid malignancies could be further incorporated in our model for solid cancers. Since the state of anti-cancer barrier systems can be measured in molecular analyses, this approach has the potential to be a bridge between epidemiology and molecular biology [53]–[55].

It is interesting to compare the results of our study with another study that used the same SEER Registry data and analyzed the factors underlying the differences between the obtained results. Recently, Rieker et al [22] described multi-step models for two ACs (colorectal and prostate) and two SCCs (laryngeal and oropharyngeal) using SEER data: the expected number of stages needed for cancer development was higher in ACs (about 10–11 for colorectal and about 23 for prostate cancer) than in SCCs (approximately about 5–6 for oropharyngeal and 7–8 for laryngeal cancers). Rieker et al [22] used the standard two-parameter multistage carcinogenesis model for a homogeneous population and applied a different approach for parameter estimation. The focus of our update in the base model generalization was on the quality of fit of age patterns of cancer incidence, especially at the region of middle and advanced ages (see Figure 2). In this region (75+ years) cancers occur at a higher rate and, probably, with rapid cancer rate growth, therefore this region is the most responsible for the precise estimation of the number of *m*-stages. The model used by Rieker et al [22] was not able to adequately describe the incidence in this region resulting in possible distortion in estimates of the number of *m*-stages. Our model is reduced to the model used by Rieker et al., if to set 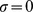 in model (1). Even in this approximation and within age region restricted by age 75, we cannot confirm their results that SCCs required lower numbers of events than ACs: e.g., our estimates show that *m*-stages for SCCs lung and esophagus were higher than respective estimates for ACs.

The existence of the “older ages” phenomenon has been confirmed by numerous demographic and epidemiological data indicating that cancer incidence (as well as cancer mortality) for most of cancers increases at a slower rate with age, leveling off around the age of 85–90 years old, and thereafter reaching a plateau and even a decline [56]–[62]. It likely mirrors one of the important breakthroughs of demography in recent years, i.e. leveling-off of the rate of oldest-old mortality rate and deviation of mortality rate from the Gompertz curve [59]. The modeling approach used in our study suggests that the observed decrease of cancer incidence at older ages could be explained by the phenomenon of “selection”: i.e., when different age groups have different susceptibility to carcinogens exposure, or different repair systems, or both. The heterogeneity in susceptibility is modeled by a frailty distribution and parameters of the distribution are estimated by applying the model to the data on incidence rates.

While interpreting the results obtained in our study, one should understand that these results could be true only if there were no other measured or latent variable(s) that could also impact the total and ACs/SCCs-specific distributions of *m*-stages. For example, the stage at cancer diagnosis could potentially be such a variable. To test the alternative hypothesis that the stage at diagnosis could affect the number of *m*-stages, correlations between the fractions of *in situ* and distant stages and the number of *m*-stages were examined. While no correlations were found when ACs and SCCs were analyzed jointly, correlations were detected in histotype-specific analysis. For example, the correlation between *m*-stages and the distant stages of cancer was r = 0.45 (p = 0.14) for twelve studied SCCs, and r = 0.47 (p = 0.17) for nine ACs originated from non-reproductive organs (i.e., excluding breast, ovarian, cervical, and prostate ACs). In pooled analysis (i.e., not ACs/SCCs-specific) this correlation disappeared (r = 0.05, p = 0.82). Histotype-specific means of *m*-stage parameter were 10.5±0.6 for SCCs and 9.3±0.5 for ACs, as well as 9.0±0.3 for ACs originated from non-reproductive organs, while the respective means of distant fractions were 10.5±2.7%, 25.2±5.6% and 27.1±5.5%, respectively. So, ACs had even higher fraction of advanced cancer stages, but still had significantly lower number of *m*-stages than SCCs. Therefore, the differences in distribution of stages at cancer diagnosis cannot explain the results presented in Figure 2. The frequency of unstaged cancers differs among cancer sites and that might be an issue that should be taken into account [63]. To check that, we compared estimated correlations between the number of *m*-stages and distant cancer stages and correlations calculated with added distant cases “hidden” among unstaged cases (assuming that the distribution of unobserved stages among the unstaged cancers was the same as the distribution of staged cancer cases for each studied year). The correlations with and without added contribution of unstaged cancers were found to be almost identical.

Certain behavioral risk factors can potentially affect some of the characteristics of carcinogenesis. However, most of population-based large datasets lack of the information on individual-based exposure to such factors. The stochastic approach in our model, which reflects hidden heterogeneity, can describe the effects of behavior factors (i.e., smoking) on predisposition/susceptibility to cancer. The exposure to such factors is one of the sources of heterogeneity modeled by the frailty distribution, i.e., parameters 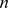 and 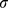 need to capture such exposure and possible their changes have to be responsible for exposure dynamics. For certain cancers the number of *m*-stages may change when certain risk factor(s) becomes more prevalent in population with time. Unfortunately, SEER registry does not provide the information on smoking and the direct study on such effects cannot be performed using this dataset. However, applying the APC analysis to our model allows indirect evaluation of smoking impact through the birth-cohort effect. Several studies adapted the TSCE model of lung carcinogenesis for a given smoking cohort and showed different effects of tobacco on cancer initiation and promotions [64], [65]. In our study, an increase for about two *m*-stages was observed from 1973–1983 to 1994–2003 for lung SCCs (but not for lung ACs) in females (see Table S1). This increase in the number of *m*-stages may be due, in part, to the change in cigarette composition over the three decades with decreased tar and nicotine coupled with the increased use of expanded and reconstituted tobacco with higher amount of 4-(methylnitrosamino)-1-(3-pyridyl)-1-butanone which may require more exposure-related events for developing of the lung SCC.

### 

#### Study limitations

Because the estimation of carcinogenesis characteristics obtained within our model assumed carcinogenesis to be a multistage process, a dependence on this assumption is a limitation. However, we reviewed and tested a spectrum of different models of carcinogenesis based on different assumptions and found that the chosen model adequately described the age patterns of incidence rates (Figure 1). That was confirmed in a sensitivity analysis. Several model assumptions can be considered as limiting factors. The assumption about population homogeneity for carcinogenesis parameters 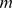 and 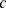 is typical for all compartmental models. In our analyses, certain cancers could be described with a better fit using a mixed model (such as two-disease models with different model parameters for two population subgroups). The underlying heterogeneity in these groups could not be captured in the current study by simple AC/SCC grouping. An inclusion of further tumor classifications (such as grade-specific, receptor-specific, and molecular pathway-specific) could decrease tumor heterogeneity, allowing a simpler one-disease model to be applied to each subgroup. Another concern is the stage at diagnosis, which was not explicitly incorporated into the model: our modeling approach was applied for cancer cases without stratification by stage at diagnosis. However, correlations between the fractions of *in situ*, distant, and distant plus unstaged stages and number of *m*-stages were studied. Our approach permits using our model parameters to compare similarities or differences in the underlying mechanisms of both common and rare cancers. For rare cancers, the model could be improved in future research by the fixation of certain parameters using auxiliary information from epidemiologic and/or molecular studies. Because our model, like other models of carcinogenesis, does not provide explicit biological interpretations of *m*-stages, the obtained results have to be carefully interpreted. Also, the lack of exposure information in SEER Registry data (such as cigarette smoking in relation to lung cancer) limits the modeling effort and requires further validation on datasets where information on specific exposures is available.

In summary, a model capable of representing the average number of stochastic events (which we denoted *m*-stages) occurring in cells during the person’s life was developed and validated using a large population dataset on cancer incidence. The numbers of *m*-stages in the model were estimated for fourteen ACs and twelve SCCs. It was found that ACs and SCCs may require different numbers of events for cancer development that may be more specific to subtype (AC, SCC) than the organ/site. The obtained results allow for developing the biomedical interpretations of this phenomenon and formulate new hypotheses that will be important for basic medical science and broad clinical applications.

## Supporting Information

Table S1 **Modeling results (i.e. fitting parameters with SE) for selected cancer histotypes model fitting, for male and female white and African-American (AAs) U.S. population for three time periods: 1973–1983, 1984–1993, and 1994–2003.**
(DOC)
