# Supplementary material for: Evaluating the Number of Stages in Development of Squamous Cell and Adenocarcinomas across Cancer Sites Using Human Population-Based Cancer Modeling
Source: PLoS One. 2012 May 22;7(5):e37430. doi: 10.1371/journal.pone.0037430 (PMC3358315; doi:10.1371/journal.pone.0037430)
Supplement: Table S1 — Modeling results (i.e. fitting parameters with SE) for selected cancer histotypes model fitting, for male and female white and African-American (AAs) U.S. population for three time periods: 1973–1983, 1984–1993, and 1994–2003. (DOC) [file pone.0037430.s001.doc]

**Supplemental table 1S.**Table S1. Modeling results (i.e. fitting parameters with SE) for selected cancer histotypes model fitting, for male and female white and African-American (AAs) U.S. population for three time periods: 1973-1983, 1984-1993, and 1994-2003

| **Cancer** | **Race** | **Sex** | **Time period** | **Age min, years** | **CHI2** | ***c*** | ***m*** | ***n*** | ***σ*** |
| --- | --- | --- | --- | --- | --- | --- | --- | --- | --- |
| Lung SCC | White | M | 1973-1983 | 30 | 3.01 | 79.0±0.8 | 9.76±0.21 | 0.66±0.09 | 6.4±0.4 |
|  |  |  | 1984-1993 |  | 1.93 | 78.4±0.6 | 10.90±0.20 | 0.85±0.06 | 7.5±0.4 |
|  |  |  | 1994-2003 |  | 1.34 | 83.2±0.4 | 10.91±0.13 | 0.64±0.05 | 7.6±0.2 |
|  |  | F | 1973-1983 |  | 1.10 | 95.6±1.5 | 8.65±0.20 | 0.38±0.07 | 14.2±0.6 |
|  |  |  | 1984-1993 |  | 1.89 | 88.9±1.1 | 10.28±0.25 | 0.51±0.08 | 11.8±0.6 |
|  |  |  | 1994-2003 |  | 1.60 | 88.9±0.7 | 11.03±0.19 | 0.50±0.06 | 11.2±0.4 |
|  | AAs | M | 1973-1983 |  | 2.12 | 66.9±1.4 | 11.72±0.62 | 0.97±0.08 | 7.8±0.9 |
|  |  |  | 1984-1993 |  | 2.06 | 69.3±1.3 | 12.29±0.58 | 1.05±0.08 | 7.9±0.9 |
|  |  |  | 1994-2003 |  | 2.53 | 77.9±1.5 | 10.80±0.43 | 0.87±0.21 | 6.8±1.0 |
|  |  | F | 1973-1983 |  | 0.63 | 79.0±4.6 | 10.25±0.95 | 0.97±0.10 | 21.9±3.5 |
|  |  |  | 1984-1993 |  | 0.96 | 82.9±1.6 | 10.29±0.40 | 0.46±0.10 | 10.9±0.8 |
|  |  |  | 1994-2003 |  | 0.90 | 88.6±1.5 | 10.07±0.31 | 0.48±0.13 | 10.1±0.7 |
| Lung AC | White | M | 1973-1983 | 30 | 1.78 | 85.4±1.5 | 8.63±0.23 | 0.76±0.09 | 10.2±0.7 |
|  |  |  | 1984-1993 |  | 1.46 | 81.1±0.8 | 9.47±0.18 | 0.83±0.06 | 8.8±0.4 |
|  |  |  | 1994-2003 |  | 1.24 | 85.2±0.5 | 9.41±0.10 | 0.59±0.06 | 7.1±0.2 |
|  |  | F | 1973-1983 |  | 1.60 | 88.6±2.3 | 8.30±0.30 | 0.77±0.06 | 17.0±1.0 |
|  |  |  | 1984-1993 |  | 2.21 | 82.7±1.4 | 9.18±0.25 | 0.77±0.06 | 11.7±0.6 |
|  |  |  | 1994-2003 |  | 1.48 | 88.0±0.7 | 8.79±0.11 | 0.58±0.05 | 8.5±0.3 |
|  | AAs | M | 1973-1983 |  | 1.41 | 68.4±2.1 | 11.03±0.75 | 0.91±0.07 | 13.4±1.3 |
|  |  |  | 1984-1993 |  | 1.03 | 71.0±1.6 | 10.18±0.44 | 0.90±0.07 | 9.4±0.8 |
|  |  |  | 1994-2003 |  | 1.73 | 72.7±1.3 | 10.95±0.40 | 1.06±0.06 | 10.7±0.9 |
|  |  | F | 1973-1983 |  | 0.98 | 57.7±2.9 | 18.25±2.55 | 1.07±0.02 | 42.7±5.8 |
|  |  |  | 1984-1993 |  | 1.08 | 71.3±2.3 | 11.10±0.67 | 0.99±0.05 | 17.1±1.7 |
|  |  |  | 1994-2003 |  | 1.56 | 84.8±2.5 | 8.82±0.40 | 0.77±0.14 | 10.8±1.2 |
| Stomach AC | White | M | 1973-1983 | 30 | 1.19 | 97.8±3.6 | 8.05±0.36 | 2.11±0.28 | 13.3±3.1 |
|  |  |  | 1984-1993 |  | 0.68 | 107.1±1.8 | 7.31±0.16 | 1.08±0.49 | 7.8±1.1 |
|  |  |  | 1994-2003 |  | 0.88 | 115.6±0.9 | 6.86±0.07 | 0.00±0.00 | 6.3±0.2 |
|  |  | F | 1973-1983 |  | 1.14 | 135.3±3.5 | 6.43±0.18 | 0.00±0.00 | 4.9±1.4 |
|  |  |  | 1984-1993 |  | 0.94 | 140.7±3.3 | 6.28±0.15 | 0.00±0.00 | 3.6±2.1 |
|  |  |  | 1994-2003 |  | 1.58 | 160.1±2.0 | 5.59±0.08 | 2.79±0.00 | 0.1±0.0 |
|  | AAs | M | 1973-1983 |  | 0.79 | 96.3±5.0 | 7.23±0.49 | 0.71±1.13 | 6.3±2.4 |
|  |  |  | 1984-1993 |  | 1.25 | 103.9±2.7 | 6.81±0.26 | 0.00±0.00 | 4.8±0.5 |
|  |  |  | 1994-2003 |  | 0.91 | 106.1±1.9 | 6.99±0.18 | 0.00±0.00 | 4.9±0.4 |
|  |  | F | 1973-1983 |  | 1.03 | 112.2±4.8 | 7.54±0.48 | 0.00±0.00 | 8.3±0.9 |
|  |  |  | 1984-1993 |  | 1.06* | 133.2±3.6 | 6.21±0.23 | 1.00±0.00 | 0.1±0.0 |
|  |  |  | 1994-2003 |  | 1.02 | 133.7±2.3 | 6.21±0.14 | 0.00±0.00 | 0.1±0.0 |
| Esophagus SCC | White | M | 1973-1983 | 30 | 0.69 | 88.9±1.7 | 11.65±0.39 | 0.87±0.07 | 23.9±1.6 |
|  |  |  | 1984-1993 |  | 1.11 | 95.0±3.2 | 10.70±0.55 | 0.89±0.12 | 25.3±2.9 |
|  |  |  | 1994-2003 |  | 1.09 | 103.5±3.2 | 10.07±0.41 | 1.03±0.15 | 29.0±3.5 |
|  |  | F | 1973-1983 |  | 0.96 | 83.0±3.1 | 14.75±1.10 | 1.04±0.04 | 58.6±5.7 |
|  |  |  | 1984-1993 |  | 0.92 | 82.5±3.7 | 15.70±1.45 | 1.16±0.04 | 64.5±8.2 |
|  |  |  | 1994-2003 |  | 1.50 | 102.8±4.5 | 11.83±0.75 | 0.95±0.18 | 42.1±6.9 |
|  | AAs | M | 1973-1983 |  | 1.08 | 66.3±1.9 | 12.65±0.90 | 0.92±0.05 | 15.6±1.4 |
|  |  |  | 1984-1993 |  | 1.00 | 72.8±2.1 | 11.19±0.65 | 0.91±0.08 | 13.6±1.4 |
|  |  |  | 1994-2003 |  | 1.69 | 73.6±2.7 | 13.26±0.98 | 1.05±0.07 | 22.3±3.2 |
|  |  | F | 1973-1983 |  | 0.95 | 76.2±3.4 | 11.55±1.00 | 0.59±0.09 | 23.4±2.2 |
|  |  |  | 1984-1993 |  | 0.67 | 83.6±3.1 | 11.19±0.76 | 0.67±0.10 | 21.1±2.1 |
|  |  |  | 1994-2003 |  | 1.33 | 101.3±5.2 | 9.33±0.64 | 0.37±0.27 | 19.2±3.2 |
| Esophagus AC | White | M | 1973-1983 | 30 | 1.16 | 91.1±11.1 | 11.53±1.84 | 1.28±0.08 | 89.1±23.9 |
|  |  |  | 1984-1993 |  | 1.17 | 103.8±4.4 | 9.15±0.48 | 0.98±0.18 | 26.8±3.9 |
|  |  |  | 1994-2003 |  | 1.03 | 100.2±2.4 | 8.79±0.27 | 1.07±0.10 | 20.5±1.8 |
|  |  | F | 1973-1983 |  | 0.74 | 129.1±8.7 | 11.82±1.27 | 0.00±0.00 | 82.3±4.2 |
|  |  |  | 1984-1993 |  | 1.00* | 118.1±12.0 | 12.45±1.89 | 1.00±0.00 | 74.7±14.6 |
|  |  |  | 1994-2003 |  | 0.83 | 129.7±9.0 | 9.06±0.66 | 1.16±0.32 | 50.3±11.1 |
|  | AAs | M | 1973-1983 |  | 0.22 | 112.3±71.4 | 15.68±19.04 | 0.00±0.00 | 134.9±53.8 |
|  |  |  | 1984-1993 |  | 0.53 | 113.0±61.9 | 11.29±8.00 | 0.68±0.93 | 113.8±96.4 |
|  |  |  | 1994-2003 |  | 1.12 | 115.1±6.7 | 10.41±0.99 | 0.00±0.00 | 33.4±2.2 |
|  |  | F | 1973-1983 |  | n/a* | 400.0±0.0 | 6.42±1.09 | 0.00±0.00 | 0.1±0.0 |
|  |  |  | 1984-1993 |  | n/a* | 10.0±0.0 | 1.00±0.00 | 0.94±0.07 | 240.0±0.0 |
|  |  |  | 1994-2003 |  | n/a* | 104.4±28.6 | 13.55±5.22 | 0.94±0.24 | 240.0±0.0 |
| Colon AC | White | M | 1973-1983 | 30 | 1.10 | 84.9±0.2 | 7.71±0.05 | 0.00±0.00 | 2.6±0.0 |
|  |  |  | 1984-1993 |  | 2.15 | 81.1±0.7 | 8.39±0.14 | 1.18±0.24 | 3.6±0.3 |
|  |  |  | 1994-2003 |  | 3.39 | 85.4±0.6 | 7.81±0.11 | 0.60±0.29 | 3.4±0.3 |
|  |  | F | 1973-1983 |  | 1.43 | 90.6±1.0 | 6.97±0.12 | 0.34±0.87 | 2.9±0.4 |
|  |  |  | 1984-1993 |  | 1.38 | 88.1±1.1 | 7.54±0.15 | 1.87±0.50 | 4.1±0.7 |
|  |  |  | 1994-2003 |  | 1.29 | 93.9±0.3 | 7.01±0.04 | 0.00±0.00 | 2.8±0.1 |
|  | AAs | M | 1973-1983 |  | 1.36 | 83.9±4.0 | 7.47±0.56 | 2.48±0.89 | 5.0±2.4 |
|  |  |  | 1984-1993 |  | 1.20 | 78.0±2.0 | 8.24±0.36 | 1.71±0.34 | 5.2±1.2 |
|  |  |  | 1994-2003 |  | 1.37 | 78.1±1.3 | 8.35±0.26 | 1.44±0.20 | 5.1±0.7 |
|  |  | F | 1973-1983 |  | 1.13 | 85.3±6.1 | 7.18±0.67 | 3.42±0.73 | 7.3±4.5 |
|  |  |  | 1984-1993 |  | 0.93 | 83.6±1.8 | 7.42±0.26 | 1.46±0.33 | 4.8±0.8 |
|  |  |  | 1994-2003 |  | 0.95 | 81.8±1.6 | 7.77±0.24 | 1.73±0.18 | 6.0±0.9 |
| Rectum AC | White | M | 1973-1983 | 30 | 0.92 | 92.7±1.4 | 8.25±0.19 | 1.04±0.16 | 8.6±0.7 |
|  |  |  | 1984-1993 |  | 1.51 | 90.3±1.7 | 8.79±0.27 | 1.19±0.13 | 10.5±1.0 |
|  |  |  | 1994-2003 |  | 1.08 | 96.7±1.2 | 7.76±0.13 | 0.94±0.11 | 9.6±0.6 |
|  |  | F | 1973-1983 |  | 0.72 | 104.7±2.3 | 7.47±0.21 | 1.07±0.23 | 10.8±1.2 |
|  |  |  | 1984-1993 |  | 1.41 | 104.8±4.4 | 7.57±0.36 | 1.65±0.32 | 13.5±3.1 |
|  |  |  | 1994-2003 |  | 0.78 | 118.3±2.4 | 6.54±0.14 | 1.04±0.32 | 10.2±1.1 |
|  | AAs | M | 1973-1983 |  | 1.54 | 75.4±7.6 | 12.49±2.59 | 1.26±0.11 | 23.3±8.1 |
|  |  |  | 1984-1993 |  | 1.00 | 95.7±4.8 | 8.19±0.56 | 0.68±0.46 | 10.6±2.5 |
|  |  |  | 1994-2003 |  | 0.90 | 93.4±4.8 | 8.06±0.52 | 1.41±0.23 | 15.3±3.3 |
|  |  | F | 1973-1983 |  | 0.82 | 79.7±8.2 | 11.40±1.95 | 1.59±0.16 | 37.8±12.4 |
|  |  |  | 1984-1993 |  | 1.06 | 86.5±10.8 | 10.21±1.87 | 1.51±0.15 | 28.6±11.7 |
|  |  |  | 1994-2003 |  | 0.88 | 90.8±7.8 | 9.10±1.00 | 1.69±0.13 | 29.7±8.3 |
| Pancreas AC | White | M | 1973-1983 | 30 | 0.98 | 95.3±2.3 | 8.41±0.28 | 1.30±0.26 | 10.6±1.6 |
|  |  |  | 1984-1993 |  | 1.42 | 96.3±2.5 | 8.54±0.30 | 1.43±0.27 | 11.4±1.9 |
|  |  |  | 1994-2003 |  | 1.44 | 94.3±1.9 | 8.84±0.24 | 1.49±0.14 | 13.6±1.6 |
|  |  | F | 1973-1983 |  | 0.89 | 98.1±2.6 | 8.78±0.32 | 1.52±0.19 | 14.2±2.1 |
|  |  |  | 1984-1993 |  | 0.75 | 101.5±1.8 | 8.61±0.21 | 1.20±0.26 | 10.8±1.3 |
|  |  |  | 1994-2003 |  | 1.10 | 103.4±1.5 | 8.46±0.17 | 0.84±0.24 | 10.2±0.9 |
|  | AAs | M | 1973-1983 |  | 1.22 | 78.0±4.8 | 10.72±1.18 | 1.29±0.17 | 15.1±4.2 |
|  |  |  | 1984-1993 |  | 1.18 | 73.2±6.4 | 12.21±1.89 | 1.51±0.08 | 24.1±8.8 |
|  |  |  | 1994-2003 |  | 1.70 | 80.2±4.0 | 10.60±0.87 | 1.38±0.13 | 17.5±4.0 |
|  |  | F | 1973-1983 |  | 1.01 | 66.1±5.6 | 17.75±4.49 | 1.29±0.08 | 38.5±10.6 |
|  |  |  | 1984-1993 |  | 0.84 | 92.7±4.8 | 8.71±0.63 | 1.68±0.38 | 12.2±3.8 |
|  |  |  | 1994-2003 |  | 1.83 | 80.5±6.9 | 11.67±1.59 | 1.60±0.13 | 24.1±10.5 |
| Liver AC | White | M | 1973-1983 | 30 | 1.58 | 85.4±6.0 | 13.37±1.62 | 1.40±0.08 | 50.5±12.3 |
|  |  |  | 1984-1993 |  | 1.37 | 114.4±4.1 | 7.98±0.34 | 0.51±0.38 | 15.2±2.3 |
|  |  |  | 1994-2003 |  | 3.61 | 63.4±3.0 | 16.63±1.87 | 1.25±0.03 | 60.5±7.2 |
|  |  | F | 1973-1983 |  | 1.20* | 186.6±16.6 | 6.08±0.43 | 1.00±0.00 | 10.0±11.5 |
|  |  |  | 1984-1993 |  | 1.03 | 148.6±5.9 | 7.20±0.30 | 0.00±0.00 | 19.1±1.4 |
|  |  |  | 1994-2003 |  | 1.15 | 130.5±5.0 | 7.56±0.30 | 0.60±0.42 | 20.6±3.2 |
|  | AAs | M | 1973-1983 |  | 1.21 | 91.3±5.8 | 9.79±1.05 | 0.37±0.27 | 17.3±3.0 |
|  |  |  | 1984-1993 |  | 0.86 | 110.2±5.9 | 6.87±0.43 | 0.30±0.48 | 10.9±2.0 |
|  |  |  | 1994-2003 |  | 0.93 | 66.3±2.4 | 13.66±1.14 | 1.11±0.02 | 29.3±2.8 |
|  |  | F | 1973-1983 |  | 0.98 | 106.6±8.1 | 11.59±1.85 | 0.00±0.00 | 26.9±2.1 |
|  |  |  | 1984-1993 |  | 1.02 | 111.3±17.3 | 9.01±1.74 | 0.81±0.57 | 32.3±13.3 |
|  |  |  | 1994-2003 |  | 1.10 | 92.0±18.7 | 10.41±2.72 | 1.38±0.16 | 46.8±27.1 |
| Kidney AC | White | M | 1973-1983 | 30 | 1.17 | 97.2±3.7 | 7.40±0.33 | 1.17±0.12 | 16.4±2.0 |
|  |  |  | 1984-1993 |  | 0.88 | 94.1±2.2 | 7.54±0.22 | 1.13±0.10 | 13.1±1.1 |
|  |  |  | 1994-2003 |  | 1.43 | 97.6±1.4 | 7.02±0.13 | 0.66±0.10 | 9.2±0.5 |
|  |  | F | 1973-1983 |  | 1.00 | 133.9±5.1 | 6.04±0.22 | 0.15±0.29 | 14.0±1.4 |
|  |  |  | 1984-1993 |  | 1.09 | 119.6±4.3 | 6.44±0.23 | 0.42±0.28 | 12.6±1.5 |
|  |  |  | 1994-2003 |  | 1.00 | 120.5±2.3 | 6.11±0.12 | 0.26±0.15 | 10.6±0.6 |
|  | AAs | M | 1973-1983 |  | 0.96 | 75.8±8.5 | 10.54±2.03 | 1.12±0.10 | 26.7±6.8 |
|  |  |  | 1984-1993 |  | 1.06 | 91.4±7.6 | 7.60±0.78 | 1.04±0.34 | 12.7±3.7 |
|  |  |  | 1994-2003 |  | 1.15 | 85.9±3.6 | 8.02±0.46 | 1.10±0.14 | 13.2±1.9 |
|  |  | F | 1973-1983 |  | 0.81 | 63.7±12.9 | 14.50±5.72 | 1.20±0.09 | 81.2±29.6 |
|  |  |  | 1984-1993 |  | 1.45 | 104.1±10.2 | 7.57±0.85 | 0.97±0.37 | 18.5±5.2 |
|  |  |  | 1994-2003 |  | 0.88 | 129.0±3.6 | 5.61±0.16 | 0.00±0.00 | 9.0±0.3 |
| Breast AC 850 | White | F | 1973-1983 | 30 | 3.66 | 58.1±0.9 | 8.61±0.22 | 1.27±0.01 | 10.8±0.5 |
|  |  |  | 1984-1993 |  | 11.21 | 54.0±0.9 | 9.65±0.32 | 1.29±0.01 | 10.0±0.5 |
|  |  |  | 1994-2003 |  | 13.38 | 59.8±0.8 | 8.00±0.19 | 1.20±0.02 | 6.5±0.3 |
|  | AAs |  | 1973-1983 |  | 1.72 | 49.5±1.9 | 10.87±0.73 | 1.26±0.02 | 19.3±2.0 |
|  |  |  | 1984-1993 |  | 1.62 | 55.2±1.3 | 8.64±0.36 | 1.30±0.02 | 10.4±0.7 |
|  |  |  | 1994-2003 |  | 2.01 | 61.2±0.9 | 7.40±0.18 | 1.20±0.03 | 6.6±0.3 |
| Breast AC 852 | White | F | 1973-1983 | 30 | 2.19 | 60.4±1.9 | 12.10±0.79 | 1.10±0.01 | 34.1±2.1 |
|  |  |  | 1984-1993 |  | 5.46 | 57.4±1.8 | 13.36±1.04 | 1.15±0.01 | 27.2±2.0 |
|  |  |  | 1994-2003 |  | 5.78 | 64.9±1.4 | 10.37±0.43 | 1.16±0.01 | 15.4±0.9 |
|  | AAs |  | 1973-1983 |  | 1.35 | 71.0±11.2 | 9.32±2.15 | 0.96±0.08 | 35.5±7.7 |
|  |  |  | 1984-1993 |  | 1.06 | 55.9±3.5 | 14.68±2.22 | 1.11±0.02 | 39.3±5.3 |
|  |  |  | 1994-2003 |  | 1.49 | 65.5±2.8 | 10.60±0.81 | 1.21±0.02 | 23.0±2.4 |
| Prostate AC | White | M | 1973-1983 | 40 | 2.30 | 71.2±0.4 | 14.48±0.29 | 1.38±0.04 | 5.8±0.3 |
|  |  |  | 1984-1993 |  | 3.56 | 67.9±0.2 | 14.25±0.18 | 1.02±0.03 | 3.7±0.1 |
|  |  |  | 1994-2003 |  | 26.59 | 64.6±0.3 | 13.31±0.27 | 0.85±0.03 | 3.7±0.1 |
|  | AAs |  | 1973-1983 |  | 1.62 | 66.6±0.7 | 15.35±0.71 | 1.30±0.06 | 5.4±0.6 |
|  |  |  | 1984-1993 |  | 1.55 | 64.4±0.3 | 14.94±0.37 | 1.10±0.04 | 3.7±0.2 |
|  |  |  | 1994-2003 |  | 4.96 | 61.3±0.3 | 12.72±0.30 | 0.88±0.04 | 3.0±0.1 |
| Ovarian AC | White | F | 1973-1983 | 30 | 1.56 | 104.3±3.5 | 5.83±0.20 | 0.74±0.11 | 11.4±0.9 |
|  |  |  | 1984-1993 |  | 1.58 | 128.8±2.0 | 4.67±0.07 | 0.00±0.00 | 6.8±0.1 |
|  |  |  | 1994-2003 |  | 1.43 | 128.2±1.3 | 4.88±0.05 | 0.00±0.00 | 7.3±0.1 |
|  | AAs |  | 1973-1983 |  | 1.01 | 120.5±9.7 | 5.74±0.45 | 0.12±0.43 | 11.1±1.9 |
|  |  |  | 1984-1993 |  | 0.94 | 134.1±6.7 | 5.18±0.25 | 0.00±0.00 | 8.8±0.5 |
|  |  |  | 1994-2003 |  | 1.44 | 144.8±6.6 | 4.89±0.20 | 0.00±0.00 | 9.0±0.5 |
| Corpus uteri AC | White | F | 1973-1983 | 30 | 3.36 | 70.2±0.9 | 10.55±0.33 | 0.75±0.03 | 10.0±0.3 |
|  |  |  | 1984-1993 |  | 1.42 | 87.6±0.9 | 7.53±0.12 | 0.39±0.06 | 7.3±0.2 |
|  |  |  | 1994-2003 |  | 2.92 | 82.3±1.1 | 8.08±0.18 | 0.80±0.04 | 9.9±0.4 |
|  | AAs |  | 1973-1983 |  | 0.94 | 103.3±7.0 | 7.11±0.62 | 0.53±0.42 | 10.7±2.2 |
|  |  |  | 1984-1993 |  | 1.26 | 90.7±3.5 | 8.95±0.60 | 0.43±0.19 | 11.3±1.3 |
|  |  |  | 1994-2003 |  | 1.80 | 97.1±3.7 | 7.54±0.42 | 0.34±0.19 | 10.1±1.0 |
| Cervix uteri SCC | White | F | 1973-1983 | 15 | 4.21 | 33.4±0.5 | 11.49±0.41 | 0.95±0.01 | 21.6±0.6 |
|  |  |  | 1984-1993 |  | 4.53 | 32.0±0.5 | 9.92±0.34 | 0.91±0.01 | 17.4±0.4 |
|  |  |  | 1994-2003 |  | 2.32 | 35.4±0.8 | 10.25±0.39 | 0.97±0.00 | 34.0±0.9 |
|  | AAs |  | 1973-1983 |  | 1.54 | 29.4±0.8 | 13.89±0.89 | 1.04±0.01 | 25.8±1.4 |
|  |  |  | 1984-1993 |  | 1.63 | 30.3±1.4 | 11.53±1.03 | 1.06±0.01 | 29.3±2.1 |
|  |  |  | 1994-2003 |  | 1.23 | 37.9±3.1 | 8.98±0.95 | 1.13±0.01 | 45.0±4.1 |
| Cervix uteri AC | White | F | 1973-1983 | 30 | n/a* | 43.1±1.1 | 20.00±0.00 | 1.05±0.01 | 192.9±13.0 |
|  |  |  | 1984-1993 |  | 1.24 | 57.1±8.5 | 10.36±2.52 | 0.96±0.03 | 76.2±11.8 |
|  |  |  | 1994-2003 |  | 1.10 | 72.4±28.9 | 7.28±3.27 | 1.01±0.03 | 80.4±22.6 |
|  | AAs |  | 1973-1983 |  | n/a* | 400.0±0.0 | 2.75±0.74 | 2.04±0.75 | 240.0±0.0 |
|  |  |  | 1984-1993 |  | 1.20 | 57.5±29.6 | 13.01±11.47 | 1.04±0.06 | 140.6±81.5 |
|  |  |  | 1994-2003 |  | 1.26* | 189.1±64.0 | 5.26±1.10 | 1.00±0.00 | 51.7±15.6 |
| Larynx SCC | White | M | 1973-1983 | 30 | 1.08 | 81.8±1.3 | 9.96±0.26 | 0.89±0.04 | 16.1±0.8 |
|  |  |  | 1984-1993 |  | 0.81 | 84.7±1.1 | 9.89±0.21 | 0.89±0.04 | 15.3±0.7 |
|  |  |  | 1994-2003 |  | 1.37 | 89.2±1.1 | 9.70±0.20 | 0.81±0.05 | 16.0±0.7 |
|  |  | F | 1973-1983 |  | 1.08 | 97.8±4.7 | 9.37±0.59 | 0.69±0.08 | 37.7±3.0 |
|  |  |  | 1984-1993 |  | 0.69 | 98.9±2.4 | 9.78±0.35 | 0.54±0.06 | 29.8±1.4 |
|  |  |  | 1994-2003 |  | 1.25 | 128.1±4.2 | 7.41±0.26 | 0.09±0.14 | 23.0±1.4 |
|  | AAs | M | 1973-1983 |  | 0.82 | 79.0±2.1 | 9.59±0.46 | 0.67±0.10 | 12.5±1.1 |
|  |  |  | 1984-1993 |  | 1.27 | 73.9±2.2 | 11.46±0.66 | 0.84±0.09 | 14.1±1.6 |
|  |  |  | 1994-2003 |  | 1.00 | 73.6±1.7 | 11.93±0.53 | 1.07±0.05 | 18.9±1.6 |
|  |  | F | 1973-1983 |  | 0.65 | 73.3±7.0 | 12.99±2.23 | 0.74±0.11 | 45.5±7.8 |
|  |  |  | 1984-1993 |  | 0.86 | 100.0±6.1 | 8.67±0.67 | 0.19±0.20 | 22.5±2.7 |
|  |  |  | 1994-2003 |  | 0.89 | 107.1±4.2 | 8.43±0.42 | 0.09±0.13 | 21.0±1.6 |
| Vulvar SCC | White | F | 1973-1983 | 15 | 1.57* | 278.2±11.0 | 3.89±0.10 | 1.00±0.00 | 0.1±0.0 |
|  |  |  | 1984-1993 |  | 3.66* | 241.3±33.6 | 3.65±0.23 | 1.00±0.00 | 18.6±3.4 |
|  |  |  | 1994-2003 |  | 2.52 | 123.5±12.6 | 4.79±0.31 | 1.47±0.07 | 33.6±4.3 |
|  | AAs |  | 1973-1983 |  | 1.00 | 89.2±27.5 | 7.95±2.31 | 0.93±0.20 | 66.6±24.8 |
|  |  |  | 1984-1993 |  | 1.13 | 194.5±102.4 | 4.03±1.05 | 1.13±0.63 | 34.1±20.7 |
|  |  |  | 1994-2003 |  | 1.16 | 171.5±26.3 | 4.14±0.37 | 0.69±0.31 | 20.8±3.9 |
| Lip SCC | White | M | 1973-1983 | 15 | 1.38 | 116.7±10.7 | 6.80±0.53 | 2.14±0.50 | 21.0±9.5 |
|  |  |  | 1984-1993 |  | 1.04 | 140.8±3.6 | 6.27±0.16 | 0.00±0.00 | 11.0±0.7 |
|  |  |  | 1994-2003 |  | 1.14 | 172.8±5.9 | 5.65±0.16 | 0.00±0.00 | 9.5±1.9 |
|  |  | F | 1973-1983 |  | 1.14* | 170.1±21.3 | 7.46±0.83 | 1.00±0.00 | 37.7±13.4 |
|  |  |  | 1984-1993 |  | 0.90 | 207.2±11.3 | 6.39±0.31 | 0.00±0.00 | 0.1±0.0 |
|  |  |  | 1994-2003 |  | 0.97* | 227.5±8.8 | 5.83±0.18 | 1.00±0.00 | 0.1±0.0 |
|  | AAs | M | 1973-1983 |  | 0.16* | 258.9±380.3 | 6.76±7.38 | 1.00±0.00 | 0.1±0.0 |
|  |  |  | 1984-1993 |  | n/a* | 400.0±0.0 | 5.16±0.46 | 0.00±0.00 | 240.0±0.0 |
|  |  |  | 1994-2003 |  | 0.20 | 111.3±26.3 | 18.56±10.07 | 0.00±0.00 | 144.0±28.9 |
|  |  | F | 1973-1983 |  | n/a* | 400.0±0.0 | 6.42±0.96 | 7.00±0.00 | 0.1±0.0 |
|  |  |  | 1984-1993 |  | n/a* | 144.2±54.9 | 11.76±5.43 | 0.68±0.76 | 240.0±0.0 |
|  |  |  | 1994-2003 |  | n/a* | 127.3±4.6 | 20.00±0.00 | 0.00±0.00 | 0.1±0.0 |
| Anal SCC | White | M | 1973-1983 | 15 | 0.82* | 127.5±23.3 | 8.47±1.44 | 1.00±0.00 | 109.4±17.7 |
|  |  |  | 1984-1993 |  | 1.08* | 136.9±16.9 | 6.65±0.58 | 1.00±0.00 | 88.0±8.6 |
|  |  |  | 1994-2003 |  | 1.00 | 71.1±5.2 | 10.05±0.85 | 1.12±0.02 | 109.8±9.6 |
|  |  | F | 1973-1983 |  | 1.17 | 119.6±28.1 | 8.93±2.04 | 1.07±0.26 | 85.1±35.5 |
|  |  |  | 1984-1993 |  | 1.03 | 157.1±16.6 | 6.72±0.54 | 0.71±0.54 | 41.6±11.2 |
|  |  |  | 1994-2003 |  | 0.88 | 76.1±4.8 | 11.62±0.98 | 1.22±0.02 | 106.2±10.5 |
|  | AAs | M | 1973-1983 |  | 0.33 | 117.2±40.8 | 9.35±3.83 | 0.00±0.00 | 108.0±17.5 |
|  |  |  | 1984-1993 |  | n/a* | 51.3±7.5 | 19.36±6.76 | 0.96±0.05 | 240.0±0.0 |
|  |  |  | 1994-2003 |  | 0.77 | 71.4±8.6 | 9.66±1.42 | 0.73±0.09 | 65.8±9.0 |
|  |  | F | 1973-1983 |  | 0.44* | 113.7±45.2 | 9.47±4.41 | 1.00±0.00 | 132.8±41.7 |
|  |  |  | 1984-1993 |  | 0.65 | 175.1±68.4 | 6.12±1.64 | 0.34±0.84 | 65.4±30.2 |
|  |  |  | 1994-2003 |  | 0.59 | 98.9±10.6 | 8.92±1.08 | 0.77±0.11 | 59.8±8.6 |
| Tongue SCC | White | M | 1973-1983 | 15 | 0.69 | 108.1±3.0 | 7.79±0.26 | 0.55±0.10 | 20.1±1.3 |
|  |  |  | 1984-1993 |  | 1.13 | 105.5±3.9 | 7.80±0.32 | 0.74±0.12 | 22.1±1.9 |
|  |  |  | 1994-2003 |  | 1.30 | 101.6±3.0 | 7.78±0.27 | 0.85±0.07 | 23.1±1.6 |
|  |  | F | 1973-1983 |  | 1.48 | 89.9±5.9 | 11.49±1.13 | 0.92±0.08 | 50.5±6.8 |
|  |  |  | 1984-1993 |  | 1.10 | 145.7±9.2 | 6.47±0.36 | 0.12±0.34 | 22.6±3.0 |
|  |  |  | 1994-2003 |  | 0.98 | 148.5±10.4 | 6.13±0.32 | 1.30±0.26 | 32.7±6.0 |
|  | AAs | M | 1973-1983 |  | 0.72 | 67.6±4.0 | 13.19±1.60 | 0.94±0.06 | 37.0±4.8 |
|  |  |  | 1984-1993 |  | 1.09 | 68.5±3.3 | 13.47±1.32 | 0.91±0.06 | 32.8±4.0 |
|  |  |  | 1994-2003 |  | 0.88 | 71.2±4.5 | 13.21±1.54 | 1.03±0.05 | 40.9±6.1 |
|  |  | F | 1973-1983 |  | n/a* | 64.1±1.2 | 20.00±0.00 | 0.84±0.05 | 85.5±8.9 |
|  |  |  | 1984-1993 |  | n/a* | 70.4±7.3 | 16.21±3.89 | 0.62±0.20 | 64.7±15.5 |
|  |  |  | 1994-2003 |  | 0.80 | 82.0±17.8 | 11.72±3.47 | 1.05±0.10 | 82.2±31.1 |
| Floor of mouth SCC | White | M | 1973-1983 | 15 | 0.81 | 87.6±2.4 | 10.88±0.46 | 0.80±0.05 | 35.5±2.2 |
|  |  |  | 1984-1993 |  | 1.19 | 88.6±3.5 | 11.10±0.62 | 0.87±0.07 | 40.2±3.7 |
|  |  |  | 1994-2003 |  | 0.89 | 91.8±2.7 | 11.21±0.46 | 0.87±0.05 | 49.1±3.4 |
|  |  | F | 1973-1983 |  | 0.93 | 86.5±3.8 | 12.77±0.94 | 0.82±0.06 | 63.4±5.6 |
|  |  |  | 1984-1993 |  | 0.88 | 91.2±4.1 | 13.14±0.97 | 0.83±0.07 | 64.5±6.4 |
|  |  |  | 1994-2003 |  | 0.91 | 98.0±6.9 | 12.28±1.12 | 1.04±0.09 | 89.4±15.8 |
|  | AAs | M | 1973-1983 |  | 0.63 | 66.1±3.7 | 14.49±1.70 | 0.75±0.07 | 37.3±4.5 |
|  |  |  | 1984-1993 |  | 0.79 | 68.1±7.5 | 13.61±2.61 | 1.00±0.07 | 49.7±12.2 |
|  |  |  | 1994-2003 |  | 0.73 | 72.5±4.2 | 14.51±1.60 | 0.89±0.06 | 57.3±7.3 |
|  |  | F | 1973-1983 |  | 0.61 | 144.7±31.7 | 7.45±1.68 | 0.00±0.00 | 61.1±6.9 |
|  |  |  | 1984-1993 |  | 0.60 | 62.0±8.3 | 18.76±6.03 | 0.87±0.09 | 140.9±34.3 |
|  |  |  | 1994-2003 |  | 0.75* | 70.8±9.8 | 18.98±5.69 | 1.00±0.00 | 199.0±38.5 |
| Gum and other mouth SCC | White | M | 1973-1983 | 15 | 1.49 | 88.6±3.5 | 11.33±0.70 | 0.99±0.08 | 38.0±4.2 |
|  |  |  | 1984-1993 |  | 0.97 | 79.8±4.6 | 12.89±1.20 | 1.24±0.03 | 61.7±8.5 |
|  |  |  | 1994-2003 |  | 1.00 | 104.2±4.7 | 9.42±0.50 | 1.18±0.10 | 42.2±5.5 |
|  |  | F | 1973-1983 |  | 0.79 | 78.1±5.7 | 15.20±1.94 | 1.20±0.03 | 88.9±15.9 |
|  |  |  | 1984-1993 |  | 1.06 | 90.3±5.3 | 12.70±1.17 | 1.24±0.06 | 65.4±10.1 |
|  |  |  | 1994-2003 |  | 0.58 | 103.6±7.0 | 10.80±0.89 | 1.70±0.08 | 73.9±15.4 |
|  | AAs | M | 1973-1983 |  | 0.63 | 73.7±5.5 | 12.49±1.71 | 0.80±0.10 | 34.6±5.4 |
|  |  |  | 1984-1993 |  | 0.73 | 71.1±5.4 | 13.63±1.94 | 0.94±0.08 | 44.2±7.8 |
|  |  |  | 1994-2003 |  | 0.83 | 85.0±4.6 | 11.22±0.95 | 0.87±0.10 | 39.0±5.2 |
|  |  | F | 1973-1983 |  | 0.41 | 71.3±6.5 | 17.04±3.57 | 0.80±0.11 | 64.2±13.1 |
|  |  |  | 1984-1993 |  | 0.77 | 142.5±15.2 | 7.39±0.86 | 0.00±0.00 | 34.9±2.6 |
|  |  |  | 1994-2003 |  | 1.14* | 124.0±25.6 | 8.91±1.85 | 1.00±0.00 | 58.5±18.3 |
| Tonsil SCC | White | M | 1973-1983 | 15 | 1.15 | 81.7±4.0 | 13.22±1.10 | 0.96±0.06 | 52.2±6.2 |
|  |  |  | 1984-1993 |  | 0.92 | 92.2±3.6 | 10.03±0.50 | 0.88±0.07 | 43.0±3.6 |
|  |  |  | 1994-2003 |  | 1.18 | 77.4±2.3 | 11.56±0.52 | 0.95±0.03 | 48.3±2.7 |
|  |  | F | 1973-1983 |  | 0.70 | 96.1±4.6 | 11.11±0.78 | 0.61±0.07 | 55.3±4.2 |
|  |  |  | 1984-1993 |  | 0.56 | 101.5±4.4 | 10.59±0.58 | 0.75±0.08 | 57.8±4.9 |
|  |  |  | 1994-2003 |  | 0.73 | 129.8±7.1 | 7.96±0.41 | 0.66±0.13 | 54.6±5.4 |
|  | AAs | M | 1973-1983 |  | 0.80 | 85.3±5.8 | 10.49±1.10 | 0.24±0.26 | 24.3±4.0 |
|  |  |  | 1984-1993 |  | 0.84 | 75.8±3.9 | 11.73±1.03 | 0.50±0.11 | 26.2±2.8 |
|  |  |  | 1994-2003 |  | 0.98 | 71.9±3.1 | 13.69±1.16 | 0.86±0.05 | 39.6±3.9 |
|  |  | F | 1973-1983 |  | n/a* | 62.8±1.1 | 20.00±0.00 | 0.85±0.05 | 96.0±9.7 |
|  |  |  | 1984-1993 |  | 0.65 | 73.0±9.0 | 15.39±3.62 | 0.79±0.13 | 89.1±21.2 |
|  |  |  | 1994-2003 |  | 0.54 | 115.7±11.1 | 8.76±0.89 | 0.32±0.21 | 48.9±7.3 |
| Hypopharynx SCC | White | M | 1973-1983 | 15 | 1.00 | 81.2±2.7 | 14.33±0.93 | 0.95±0.05 | 45.8±4.3 |
|  |  |  | 1984-1993 |  | 1.02 | 84.9±2.7 | 13.13±0.69 | 0.92±0.06 | 41.9±3.9 |
|  |  |  | 1994-2003 |  | 1.36 | 103.6±3.8 | 10.19±0.46 | 0.68±0.13 | 35.9±3.8 |
|  |  | F | 1973-1983 |  | 0.84 | 87.1±5.1 | 14.39±1.47 | 0.81±0.07 | 88.7±10.2 |
|  |  |  | 1984-1993 |  | 0.68 | 102.3±5.2 | 11.75±0.84 | 0.64±0.11 | 63.0±6.5 |
|  |  |  | 1994-2003 |  | 0.85 | 119.6±8.4 | 10.24±0.83 | 0.75±0.17 | 73.6±10.9 |
|  | AAs | M | 1973-1983 |  | 1.06 | 90.1±7.3 | 9.80±1.26 | 0.25±0.25 | 23.1±3.6 |
|  |  |  | 1984-1993 |  | 0.79 | 76.4±4.0 | 12.60±1.31 | 0.79±0.09 | 30.0±3.7 |
|  |  |  | 1994-2003 |  | 1.10 | 92.3±6.4 | 10.40±1.00 | 0.74±0.20 | 30.5±5.9 |
|  |  | F | 1973-1983 |  | n/a* | 65.8±1.4 | 20.00±0.00 | 0.71±0.10 | 101.4±13.7 |
|  |  |  | 1984-1993 |  | n/a* | 71.0±1.3 | 20.00±0.00 | 0.73±0.21 | 125.2±34.1 |
|  |  |  | 1994-2003 |  | 0.61 | 112.7±20.2 | 9.90±1.89 | 0.24±0.58 | 62.3±22.8 |

Note: * - Parameter estimation was performed within linear constraints: 10≤*c*≤400, 1≤*m*≤20, 0≤*n*≤7, 0.1≤*σ*≤240. Due to the limited statistical power for certain cases the model parameters can be over these constraints: for these cases we tested three-parameter model with *n*=1, that corresponds to gamma distribution of frailty. If solution is found, it is presented in the table and marked by asterisk for the value of *χ*^2^. If the solution inside the region is not found for both models, *χ*^2^ is marked as n/a* and original four-parameter solution is presented.
